# Supplementary material for: Effects of Sodium Alginate, Pectin and Chitosan Addition on the Physicochemical Properties, Acrylamide Formation and Hydroxymethylfurfural Generation of Air Fried Biscuits
Source: Polymers (Basel). 2022 Sep 22;14(19):3961. doi: 10.3390/polym14193961 (PMC9572387; doi:10.3390/polym14193961)
Supplement: Supplementary file 1 [file polymers-14-03961-s001.zip › polymers-1902076-supplementary.pdf]

Table S1 Correlation analysis of sodium alginate concentration, acrylamide content, pH value, DPPH radical scavenging activity, reducing power and CIE color value of biscuits.

	Sodium alginate concentration	Acrylamide content	pH	DPPH radical scavenging activity	Reducing power	CIE L*	CIE a*	CIE b*	BI
Sodium alginate concentration	1								
Acrylamide content	-0.478**	1							
pH	0.532**	-0.467*	1						
DPPH radical scavenging activity	0.189	0.102	-0.394*	1					
Reducing power	0.776**	-0.106	0.379*	0.105	1				
CIE L*	0.210	-0.221	0.112	0.090	0.217	1			
CIE a*	-0.083	0.275	-0.128	0.012	0.101	-0.550**	1		
CIE b*	0.072	0.108	0.022	0.082	0.208	0.495**	0.356*	1	
BI	-0.162	0.320	-0.126	0.005	-0.034	-0.555**	0.937**	0.436**	1

* and ** indicate significance at $p < 0.05$ and $p < 0.01$ respectively.

BI: browning index.

Table S2 Correlation analysis of pectin concentration, acrylamide content, pH value, DPPH radical scavenging activity, reducing power and CIE color value of biscuits.

	Pectin concentration	Acrylamide content	pH	DPPH radical scavenging activity	Reducing power	CIE L*	CIE a*	CIE b*	BI
Pectin concentration	1								
Acrylamide content	-0.276	1							
pH	-0.654**	0.276	1						
DPPH radical scavenging activity	0.129	-0.436*	-0.300	1					
Reducing power	0.644**	-0.559**	-0.393*	0.357*	1				
CIE L*	-0.064	0.245	-0.146	0.094	0.024	1			
CIE a*	-0.076	0.250	0.297	-0.032	-0.146	-0.283	1		
CIE b*	-0.182	0.264	0.315	-0.001	-0.189	-0.403*	0.574**	1	
BI	-0.073	-0.018	0.389*	-0.039	-0.150	-0.500**	0.751**	0.576**	1

* and ** indicate significance at $p < 0.05$ and $p < 0.01$ respectively.

BI: browning index.

Table S3 Correlation analysis of chitosan concentration, acrylamide content, pH value, DPPH radical scavenging activity, reducing power and CIE color value of biscuits.

	Chitosan concentration	Acrylamide	pH	DPPH radical scavenging activity	Reducing power	CIE L*	CIE a*	CIE b*	BI
Chitosan concentration	1								
Acrylamide	-0.512**	1							
pH	0.217	0.405*	1						
DPPH radical scavenging activity	-0.167	-0.110	-0.348*	1					
Reducing power	0.491**	-0.666**	-0.486**	0.010	1				
CIE L*	0.066	-0.144	-0.080	-0.005	0.313	1			
CIE a*	0.199	0.021	0.085	0.192	0.131	-0.295	1		
CIE b*	0.088	-0.280	-0.198	0.204	0.349*	0.813**	0.136	1	
BI	0.050	-0.166	-0.145	0.329*	0.041	-0.401*	0.798**	0.200	1

* and ** indicate significance at $p < 0.05$ and $p < 0.01$ respectively.

BI: browning index.
